# Dill Extract Induces Elastic Fiber Neosynthesis and Functional Improvement in the Ascending Aorta of Aged Mice with Reversal of Age-Dependent Cardiac Hypertrophy and Involvement of Lysyl Oxidase-Like-1

**DOI:** 10.3390/biom10020173

**Published:** 2020-01-23

**Authors:** Wassim Fhayli, Quentin Boëté, Nadjib Kihal, Valérie Cenizo, Pascal Sommer, Walter A. Boyle, Marie-Paule Jacob, Gilles Faury

**Affiliations:** 1 Univ. Grenoble Alpes, Inserm, CHU Grenoble Alpes, HP2, 38000 Grenoble, France; wassimfhayli@gmail.com (W.F.); boete.quentin@gmail.com (Q.B.); 2 Laboratoire de Phytochimie et de Pharmacologie, Département de Chimie, Université de Jijel, Jijel 18000, Algeria; nadjib.kihal@icloud.com; 3 L’Occitane en Provence, 04100 Manosque, France; val.cenizo@gmail.com; 4 Institut de Biologie et Chimie des Protéines UMR5305—LBTI, CNRS, 69367 Lyon, France; pascal.sommer@univ-amu.fr; 5 Department of Anesthesiology and Critical Care Medicine Division, Washington University School of Medicine, St Louis, MO 63110, USA; boylew@wustl.edu; 6 INSERM, U1148, and Hopital Bichat-Claude Bernard, 75018 Paris, France; marie-paule.jacob@orange.fr

**Keywords:** ascending aorta, elastic fibers, dill extract, lysyl oxidase-like-1 (LOXL-1), arterial mechanics, aging

## Abstract

Elastic fibers (90% elastin, 10% fibrillin-rich microfibrils) are synthesized only in early life and adolescence mainly by the vascular smooth muscle cells through the cross-linking of its soluble precursor, tropoelastin. Elastic fibers endow the large elastic arteries with resilience and elasticity. Normal vascular aging is associated with arterial remodeling and stiffening, especially due to the end of production and degradation of elastic fibers, leading to altered cardiovascular function. Several pharmacological treatments stimulate the production of elastin and elastic fibers. In particular, dill extract (DE) has been demonstrated to stimulate elastin production in vitro in dermal equivalent models and in skin fibroblasts to increase lysyl oxidase–like-1 (LOXL-1) gene expression, an enzyme contributing to tropoelastin crosslinking and elastin formation. Here, we have investigated the effects of a chronic treatment (three months) of aged male mice with DE (5% or 10% *v/v*, in drinking water) on the structure and function of the ascending aorta. DE treatment, especially at 10%, of aged mice protected pre-existing elastic lamellae, reactivated tropoelastin and LOXL-1 expressions, induced elastic fiber neo-synthesis, and decreased the stiffness of the aging aortic wall, probably explaining the reversal of the age-related cardiac hypertrophy also observed following the treatment. DE could thus be considered as an anti-aging product for the cardiovascular system.

## 1. Introduction

Elastic fibers, composed of an elastin core (90%) surrounded by fibrillin-rich microfibrils (10%), are essential extracellular matrix (ECM) macromolecules endowing extensible tissues with critical mechanical properties such as resilience, flexibility, and elasticity. Elastic fibers, mainly arranged into concentric elastic lamellae in the arterial wall, are responsible for the main part of the elastic properties of the large arteries, i.e., the aorta and its main branches [1,2,3,4]. The mechanics of the large elastic arteries is one of the principal contributors to appropriate hemodynamics since the main role of central arteries is to cushion the oscillations of the blood pressure and flow, produced by the discontinuous ventricular ejection. During systole, the distension of the aorta and its proximal branches allows for the storage of both ~50% of the left ventricle stroke volume and energy in the extended elastic fibers [5]. During diastole, the arterial wall elastic fibers, returning to their relaxed state, release the accumulated energy, applying pressure to the blood which forwards the stored blood volume to the peripheral circulation. This maintains a relatively elevated arterial blood pressure and flow during diastole. This cushioning phenomenon, called the Windkessel effect, helps to decrease the heart afterload, smoothens the pressure changes in the arterial circulation, and generates a more continuous peripheral blood flow [4,5,6,7,8,9]. Aging and genetic deficiency in elastin or microfibrils lead to serious alterations of the arterial physiology and/or pathologies such as Williams or Marfan syndromes [4,6,10,11,12,13].

Elastin, accounting for an important part of the artery wall weight (> 50% of the dry weight of the ascending aorta wall), results from the cross-linking by lysyl-oxydase (LOX) and lysyl-oxidase-like-1 (LOXL-1) of its soluble precursor, tropoelastin. Tropoelastin is essentially synthesized by the vascular smooth muscle cells (VSMCs) only in early life and adolescence. No -or extremely limited- production of elastin and elastic fibers, i.e., elastogenesis, takes place in adult or aged human beings or animals [2,4,6,13,14,15,16,17,18]. Therefore, from their maximum quantity and integrity existing after childhood, elastin and elastic fibers are progressively degraded during aging by mechanical (fatigue) and enzymatic (e.g., matrix metalloproteases-MMPs-, cathepsins) processes [19,20,21,22]. As elastin gradually fails, its load-bearing function is taken over by collagen, with an increased collagen/elastin ratio, resulting in increased arterial stiffness and decreased arterial distensibility, as observed in the aging processes [4,23,24,25]. Several additional age-dependent mechanisms also contribute to arterial stiffening, including medial calcification and lipid deposition, which both take place, for an important part, within the elastic fibers [4,26]. Vascular stiffening causes arterial wave reflexions at the end of systole rather than during diastole, therefore increasing systolic and decreasing diastolic blood pressures, thus increasing pulse pressure [27]. The elevation of the systolic blood pressure increases the systolic workload of the left ventricle (LV), leading to increased oxygen consumption, LV hypertrophy, and interstitial fibrosis. All of these events predispose to the development of age-related heart failure [4,28].

Many therapeutical strategies, either non-pharmacological (exercise training, weight loss, or specific diets) or pharmacological (anti-hypertensive treatments, lipid-lowering agents, AGEs breakers) have been evaluated for their capacity to limit the age-related arterial stiffening. Some of these drugs have presented some positive effects by acting through a modulation of arterial fibrosis or smooth muscle cell contractility or by less understood mechanisms [28]. Recently, different pharmacological treatments targeting the enhancement of elastin production and elastic fiber assembly have been evaluated [4]. Several ATP-dependent potassium channel openers, including minoxidil, or their derivatives, have been shown, in vitro or in vivo in VSMCs or arteries of rats or mice, to promote elastic fiber neosynthesis and/or elastin LOX or LOXL-1 expressions [29,30,31,32,33,34,35,36]. Dill extract (DE) has been shown to increase skin elasticity in women [37] and, in vitro, lysyl oxidase–like-1 (LOXL-1) gene expression in skin fibroblasts and elastin production in dermal equivalent models [38]. This is of particular importance since LOXL-1 initiates covalent crosslinking of the elastin precursor (tropoelastin) molecules, which is a crucial step of normal elastin maturation [2,39]. As reported here, we have investigated the impact of a three-month treatment with DE on elastin production, protection or recovery of elastic fibers, and arterial structure and function in aged mice, which normally no longer produce elastic fibers. Our results indicate that DE treatment reverses age-related cardiac hypertrophy, protects elastic lamellae (EL), reactivates elastin and elastic fiber synthesis, and improves the biomechanical properties of the aging aortic wall.

## 2. Materials and Methods

### 2.1. Animals

Twenty-six aged C57BL6/J male mice (24–25 months of age) were used: 10 in the untreated group (control), 8 in the group treated with 5% DE, and 8 in the group treated with 10% DE (see below). In addition, two 24-month-old untreated mice were used for primary cultures of VSMCs from the ascending aorta. Ten 6 month-old untreated C57BL6/J male mice were used as adult controls regarding the heart and body weights, blood pressure, histology and vascular mechanics. All animal housing and surgical procedures were in accordance with institutional guidelines.

### 2.2. Aqueous Extract of Dill and Treatment

The pure aqueous dill extract (DE) was prepared by mixing 5 g of dill seed powder with distilled water qs 100 g. The mixture was agitated overnight at 4 °C and then centrifuged for 15 min at 8000× *g* at 4 °C. The final product, i.e., DE, was obtained after four successive filtration steps (3, 1.2, 0.8, and 0.45 µm) [37].

A 3-month chronic treatment with two concentrations of DE (5% or 10% *v/v*) in drinking (tap) water was administered to the aged mice (21–22 months at the beginning of treatment, 24–25 months at the end of treatment). The DE-supplemented water was replaced every two days. Several successive batches of DE were used throughout the 3-month treatment period. Control mice were given tap water without DE.

### 2.3. Blood Pressure

Blood pressure was measured at the tail artery in awake animals by using a CODA tail-cuff recorder (Kent Scientific, Torrington, CT, USA). Measurements were repeated two times a day for the 3 last days of treatment. The values obtained on days 2 and 3 were averaged for each animal, as previously described [33].

### 2.4. Surgical and Post-Surgical Procedures

At the end of the in vivo study period and following the blood pressure measurements, all experimental and control mice were anesthetized using pentobarbital (60 mg/kg) for organ collection. The organs were then studied by using the following methods.

#### 2.4.1. Heart Weight

Hearts were collected, washed, and weighed (wet weight). Left ventricle, right ventricle, and septum were then dissected, washed, and separately weighed (wet weight). Total heart weight to body weight (HW/BW), left ventricle + septum weight to body weight (LV + S/BW), and right ventricle weight to body weight (RV/BW) ratios were calculated as percentages.

#### 2.4.2. Ascending Aorta Mechanics and Reactivity

The ascending aorta was excised and placed in a physiological buffer composed of 135 mM NaCl, 5 mM KCl, 1.6 mM CaCl_2_, 1.17 mM MgSO_4_, 0.44 mM KH_2_PO_4_, 2.6 mM NaHCO_3_, 0.34 mM Na_2_HPO_4_, 5.5 mM D-glucose, 0.025 mM EDTA, 10 mM HEPES (pH 7.4). The vessels were then cannulated and mounted onto a pressure myograph placed under a video microscope, allowing for aorta bathing and filling with a physiological solution at 37 °C. A proprietary video analysis software system, WinDiam, coupled to the video microscope, was used to measure the inner and outer vessel diameters while changing the intraluminal pressure of the vessel, i.e., changing the pressure of the physiological buffer filling the vessel lumen from 0 to 175 mmHg. Below 125 mmHg, the inner diameter was calculated as described [12,40].

Distensibility, i.e., the change in relative luminal volume (percentage) per mmHg [41], was then calculated. Here, we have used the distensibility per 25-mmHg increment (D_25_). In the following, “distensibility” will mean D_25_.

Circumferential midwall strain (ε), circumferential wall stress (σ), and incremental elastic modulus (Einc) were calculated according to classical formulas [42]. ε is relative increase in diameter, at a given pressure, as compared to the diameter at no pressure. σ are the forces that are circumferentially applied on each small portion (surface) of the vessel wall. Einc is indicative of wall stiffness.

Variations of the ascending aorta diameter in response to 10^−5^ M phenylephrine (PE), a VSMC-dependent vasoconstrictor mainly acting through the alpha1-adrenoceptors, then 10^−5^ M acetylcholine (Ach), an endothelial cell-dependent vasodilator, was assessed at 75 mmHg [12].

The detailed protocol is described in Appendix A.

#### 2.4.3. Histological Examination

The ascending aorta was excised and conserved in 4% PFA for one night at 4 °C and embedded in paraffin. Five-micrometer sections were cut, deparaffinized with xylene, hydrated in ethanol, and then rinsed with water. Weigert coloration (resorcin-fuschin) was used for the staining of elastic fibers. After staining, the ascending aorta cross-sections were examined under a light microscope (Nikon, France). The number of elastic lamellae and elastic lamellae disruptions were counted in high magnification images (40 × objective) from three animals per group. For these experiments, in 3 separate ascending aorta cross-sections from each animal, the numbers of elastic lamellae were counted in 8 distinct locations of the wall (~ every 45°) and elastic lamellae disruptions were counted in the entire aorta wall. The counts of elastic lamellae and the ratio of lamellae disruptions to total lamellae were then averaged and compared between the groups.

#### 2.4.4. RNA Analyses

Total RNA was extracted from the thoracic descending aorta with the E.Z.N.A.^®^ total RNA kit I (Omega Biotek, Inc., Norcross, GA, USA) and genomic DNA was digested with DNase I. Gene expression levels were evaluated by real-time PCR using a CFX96^®^ real-time system with the IQ™ SYBR^®^ Green supermix (Bio-Rad, Marnes-la-Coquette, France) after oligodT- primed reverse transcription of 200 ng of total RNA. Expression of tropoelastin (TE) and lysyl-oxidase like-1 (LOXL-1) mRNAs were normalized against total mRNA levels, quantified using the Quant-iT™ Ribogreen^®^ RNA assay kit (InVitrogen, Cergy-Pontoise, France). Amplification primers for the tested gene were 5′-AAGCTGCTGCTAAGGCTGC-3′ (antisense) and 5′-TGCAACTCCTCCACCTGGGAA-3′ (sense) for tropoelastin, and 5′-CAGCTTCTGCCTGGAGGACA-3′ (antisense) and 5′-CGTAGCGACCTGTGTAGTGGATG-3′ (sense) for lysyl-oxidase like-1 [13,33].

#### 2.4.5. Elastin Production by Cultured VSMCs

##### Cell Culture

As previously described [36], vascular smooth muscle cells (VSMCs) were isolated from the ascending aortae of two 24-month-old mice by enzymatic digestion with collagenase type 2 (1 mg/mL) and elastase (0.5 mg/mL) for 40 min at 37 °C. The suspension was centrifuged at 600 × *g* for 10 min, and the cells were collected and seeded in 2 wells from a 48-wells plate (cells from each ascending aorta in a separate well) in Dulbecco’s modified Eagle’s medium (DMEM), containing 20% bovine fetal serum (FBS), 1% (*v/v*) penicillin/streptomycin solution and 1% non-essential amino acids solution (NEAA), and maintained in 5% CO_2_ humidified air at 37 °C. After confluence, cells were isolated by trypsinization, cell culture was amplified and used at the 4th passage.

##### Extracellular Elastin Quantification

As previously described [36], VSMCs were cultured in 96-well plates in fresh 1% FBS-DMEM until they reach total confluency. They were then bathed in 1% FBS-DMEM supplemented with 0%, 1%, or 3% DE (*v/v*, final concentration). Because, in cell cultures, DE does not undergo the digestion process and the associated potential loss of potency (in contrast to the chronic DE treatment of mice), the DE concentrations used for cultured VSMC treatment were lower than those used in the chronic treatment of mice, in accordance with DE concentrations previously used in skin cell cultures [38]. The effects of the corticosteroid dexamethasone (0.1 µM) and the ATP-dependent potassium channel opener diazoxide (50 µM) were also tested as potential positive controls, with previously described stimulation of elastin expression by fibroblasts or VSMCs [32,34,36,43]. After 48 h, VSMCs were exposed to the primary antibody to elastin (ab21610, Abcam, Paris, France) before application of the secondary antibody coupled to horseradish peroxidase (HRP). This was followed by addition of the substrate of HRP, 3,3′,5,5′-tetramethybenzidine (TMB), the reaction being stopped by the addition of sulfuric acid. The reaction end-product was then quantified by measuring its absorbance at 450 nm, which was calibrated to the elastin concentration in the well.

#### 2.4.6. Statistics

Comparisons were done using one- or two-way ANOVA followed when necessary by Fisher’s least significant difference test (LSD) for paired value comparisons. Unless otherwise indicated, the results are presented as mean values ± SEM, and *p*-values ≤ 0.05 were considered as statistically significant.

## 3. Results

### 3.1. Blood Pressure

**Figure 1:**
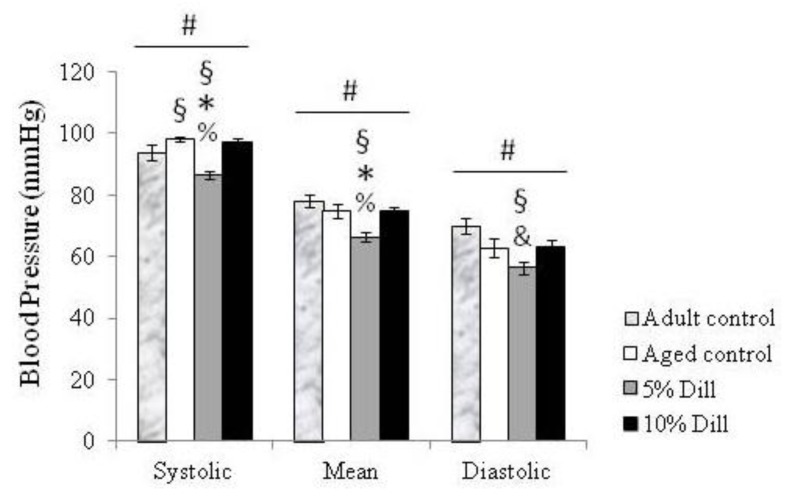
Systolic, mean, and diastolic arterial blood pressure in control (untreated) and DE-treated aged mice. Values are mean ± SEM. Control: untreated animals. Dill: DE-treated animals. ^#^ Significant effect of treatment (one-way ANOVA, *p* ≤ 0.05). ^§^ Significant difference with untreated adult mice (LSD test following one-way ANOVA, *p* ≤ 0.05). * Significant difference with untreated aged mice (LSD test following one-way ANOVA, *p* ≤ 0.05). ^%^ Significant difference with 10% DE-treated aged mice (LSD test following one-way ANOVA, *p* ≤ 0.05). ^&^ Strong trend towards a difference between 5% DE-treated mice and corresponding untreated adult mice as well as 10% DE-treated aged mice (LSD test following one-way ANOVA, *p* = 0.06). n = 4–6 in each group.

Systolic blood pressure was lower in adult compared to aged untreated animals. Treatment with 5% DE induced a significant 11–12% decrease in systolic and mean blood pressures of aged animals, as compared to both untreated and 10% DE-treated aged mice. A similar strong trend was observed regarding diastolic blood pressure. Systolic, mean, and diastolic blood pressures were also significantly lower in 5% DE-treated aged mice compared to untreated adult animals. Treatment of aged mice with 10% DE did not significantly change blood pressure compared to untreated aged animals or untreated adult animals (Figure 1).

### 3.2. Body and Heart Weight Measurements

Body weight was not significantly changed by DE treatments nor by aging. Total heart weight-, left ventricle plus septum weight-, and right ventricle to body weight ratios (HW/BW, LV + S/BW and RV/BW, respectively) were measured in all groups of mice. Interestingly, the treatments with 5% DE and 10% DE completely reversed the age-dependent cardiac hypertrophy observed in untreated animals by inducing HW/BW and LV + S/BW ratio decreases in the range of 15% in treated aged animals. RV/BW ratios seemed to be unaffected by age and treatment (Table 1).

### 3.3. Ascending Aorta Morphology

**Figure 2:**
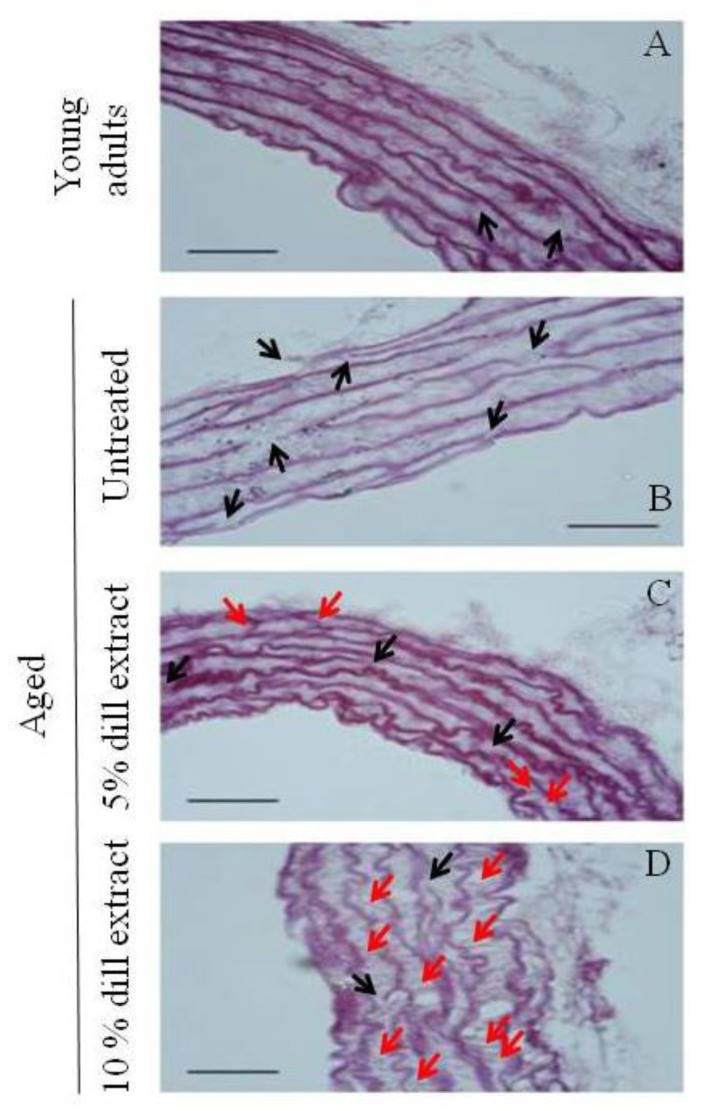
Histology of the ascending aorta from (**A**) untreated adult mice, (**B**) untreated aged mice, (**C**) 5% DE-treated aged mice, (**D**) 10% DE-treated aged mice. Cross-sections with Weigert staining of the elastic fibers. Bar = 50 µm. Black arrows: elastic lamella disruptions. Red arrows: neo-synthesized elastic fibers. n = 3 animals per group.

Weigert staining showed that, compared to untreated adult and aged mice (Figure 2A,B), additional neo-synthesized elastic fibers of various orientations were observed in the ascending aorta wall of DE-treated aged mice. This was particularly evident in 10% DE-treated mice, in which many of these neo-elastic fibers were present, some of them being radially-oriented, i.e., bridging the pre-existing elastic lamellae (Figure 2C,D).

As compared to untreated adult animals, the aortic elastic lamellae (EL) of untreated aged animals appeared considerably fragmented, while treatment of aged animals with DE resulted in more continuous EL, with significantly less fragmentations compared to untreated controls. A trend towards less disruptions at 10% DE compared to 5% DE was also observed. The number of disruptions of each elastic lamella of aged mice was reduced by 23% after 5% DE treatment and by 33% after 10% DE treatment. The number of EL in the media of the ascending aorta was not significantly affected by age or treatment (Table 2).

### 3.4. Tropoelastin and Lysyl-Oxidase Like-1 mRNA Levels

**Figure 3:**
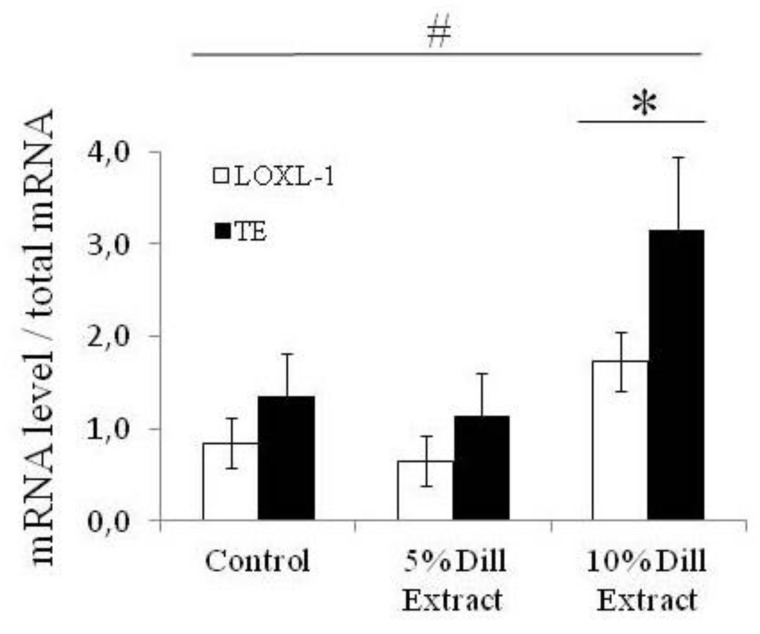
Tropoelastin (TE) and lysyl-oxidase-like-1 (LOXL-1) mRNA levels after DE treatments of aged mice. ^#^ Significant effect of treatment (two-way ANOVA, *p* ≤ 0.05). * Significant difference between 10% DE-treated mice and corresponding untreated (control) 24-month-old mice (LSD test following two-way ANOVA, *p* ≤ 0.05). n = 3–7 in each group.

Tropooelastin (TE) and lysyl-oxidase-like-1 (LOXL-1) gene expressions were quantified by measurement of the mRNA levels. Chronic treatment with DE had a general significant effect on TE and LOXL-1 gene expressions. Treatment with 10% DE, not 5% DE, produced a substantial elevation of both TE and LOXL-1 mRNA levels, in the range of a doubling, compared to controls (Figure 3).

### 3.5. Biomechanical Properties of the Ascending Aorta

**Figure 4:**
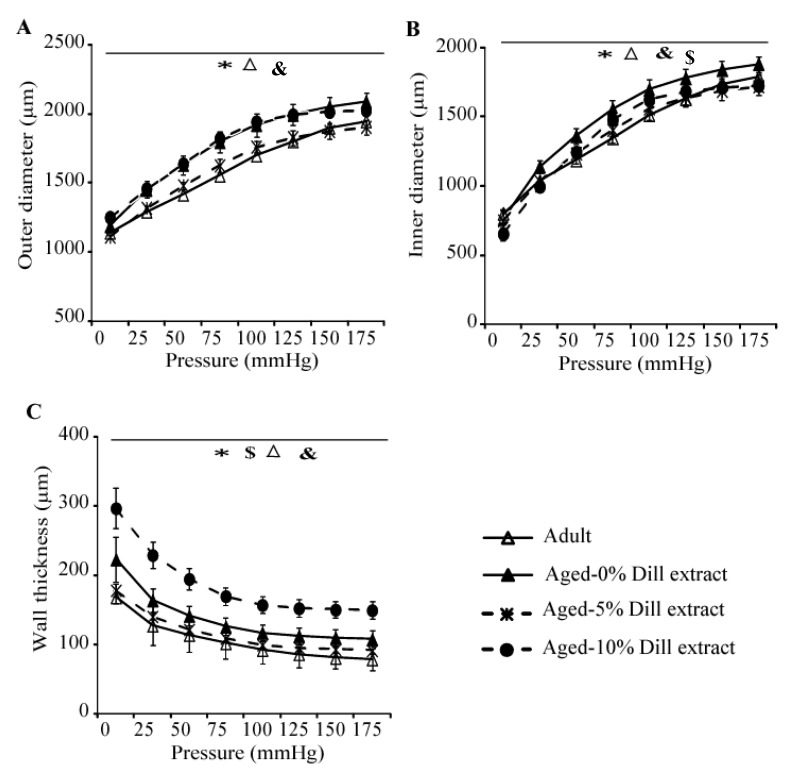
Diameter-pressure curves and wall thickness of the ascending aorta of untreated, 5% DE- or 10% DE-treated aged mice, as well as untreated (control) adult animals. (**A**) Outer diameter-pressure relation; (**B**) inner diameter-pressure relation; (**C**) wall thickness-pressure relation. * General significant difference between untreated adult and untreated aged mice (two-way ANOVA, *p* ≤ 0.05). ^&^ General significant difference between 10% DE-treated aged mice and untreated adult mice (two-way ANOVA, *p* ≤ 0.05). ^$^ General significant difference between 10% DE-treated and untreated aged mice (two-way ANOVA, *p* ≤ 0.05). ^Δ^ General significant difference between 5% DE-treated and untreated aged mice (two-way ANOVA, *p* ≤ 0.05). n = 4–7 per group.

Ex-vivo biomechanical studies of the ascending aorta exhibited a general age-dependent increase in arterial outer diameter (OD), inner diameter (ID), and wall thickness (Figure 4A–C). Treatment with 5% DE induced a reduction of OD, ID, and wall thickness, compared to those of untreated aged mice. Therefore, the dimensions of 5% DE-treated arteries of aged animals returned closer to those of the untreated adult animals (Figure 4A–C). It was also observed that 10% DE treatment of aged mice induced a thickening of the arterial wall (Figure 4C), leading in particular to a reduced ID compared to age-matched untreated animals (Figure 4B).

**Figure 5:**
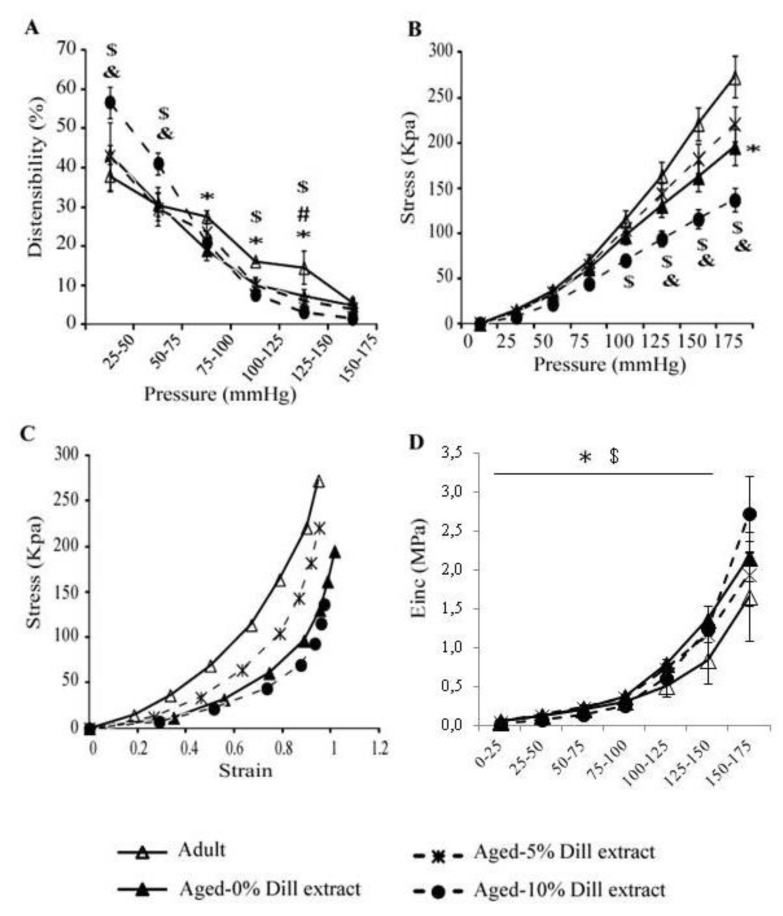
Mechanical parameters of the ascending aorta of untreated 5% DE- or 10% DE-treated aged mice, as well as untreated (control) adult animals. (**A**) Aortic distensibility-pressure increment relation; (**B**) circumferential stress-pressure relation; (**C**) stress-strain relation; (**D**) incremental elastic modulus (Einc)-pressure increment relation. ^*^ Significant difference between untreated adult and untreated aged mice (two-way ANOVA followed by LSD test if necessary, *p* ≤ 0.05). ^&^ Significant difference between 10% DE-treated aged mice and untreated adult mice (two-way ANOVA followed by LSD test if necessary, *p* ≤ 0.05). ^$^ Significant difference between 10% DE-treated aged mice and untreated aged mice (two-way ANOVA followed by LSD test if necessary, *p* ≤ 0.05). ^#^ Significant difference between 5% DE-treated mice and adult control mice (two-way ANOVA followed by LSD test if necessary, *p* ≤ 0.05). n = 4–7 per group.

Regarding the mechanical parameters, a general age-dependent decline in distensibility was observed in the 75–150 mmHg pressure range (Figure 5A). No significant improvement of the aortic distensibility was observed in aged mice after 5% DE treatment (Figure 5A). However, aortae from 10% DE-treated aged animals presented an increased distensibility at low pressure (25–75 mmHg, covering in part the physiological pressure range), compared to adult and aged untreated animals (Figure 5A). Circumferential wall stress, inversely proportional to wall thickness, was found lower at high pressure in untreated aged animals compared to corresponding untreated adult mice. After 10% DE treatment of aged animals, circumferential stress was also found significantly lower in the 125–175 and 100–175 mmHg pressure ranges, when compared to untreated young and aged animals, respectively (Figure 5B). The aorta stress-strain curve from 5% DE-treated mice was between those from untreated adult and aged animals. At similar circumferential strain, the arterial wall stress values of 10% DE-treated aged mice were lower than that of adult and, to a lower extent, aged untreated animals (Figure 5C). Incremental elastic modulus (Einc), indicative of the wall material stiffness, was increased by aging in the untreated groups, as expected. Interestingly, when compared in the 0–150 mmHg range, the aorta Einc was significantly decreased in 10% DE-treated aged mice. In this group, Einc values were lower than those from age-matched untreated animals and close to those from untreated adult mice. In the 150–175 mmHg range, no significant differences between groups could be observed, possibly because of the higher variability of Einc and markedly increased aortic Einc in 10% DE-treated mice, reaching their maximal strain in this range (compensating their higher distension at lower pressures) (Figure 4A,B and Figure 5D).

### 3.6. Ex Vivo Response of Ascending Aorta Segments to Vasoactive Agents

**Figure 6:**
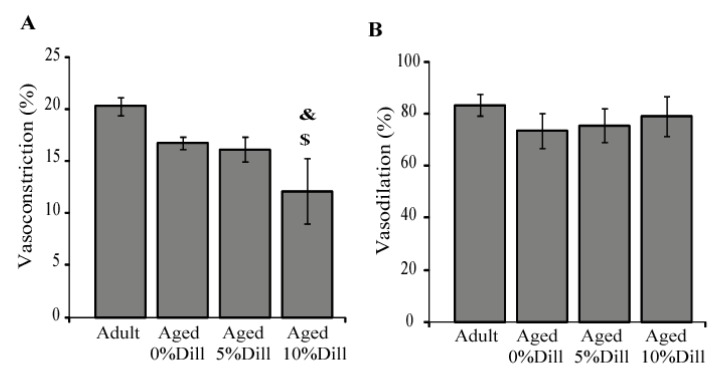
Influence of 5% and 10% DE treatment on the ascending aorta reactivity in aged mice, compared to adult or aged untreated animals. (**A**) Response to the vasoconstrictor phenylephrine (10^−5^ M) on the arterial inner diameter; (**B**) response to the vasodilator acetylcholine (10^−5^ M) on the restoration of the inner diameter of the aorta pre-constricted with 10^−5^ M phenylephrine. Dill: DE. ^&^ Significant difference between 10% DE-treated aged mice and untreated adult (control) mice (one-way ANOVA followed by LSD tests, *p* ≤ 0.05). ^$^ Significant difference between 10% DE-treated aged mice and untreated aged (control) mice (one-way ANOVA followed by LSD test, *p* ≤ 0.05). n = 4–7 per group.

A strong trend towards an age-dependent decrease in the phenylephrine (PE)-induced vasoconstriction was observed in untreated animals: the aortic diameter decrease was of 17% in aged mice compared to 20% in adult animals (one-way ANOVA, *p* = 0.06; Figure 6A). Interestingly, when compared with adult and aged controls, chronic treatment with 10% DE, not 5% DE, reduced the vascular response to phenylephrine (Figure 6A). Regarding the response to acetylcholine (Ach), no significant difference between groups could be detected (Figure 6B).

### 3.7. Impact of DE on Elastin Production by Cultured VSMCs

**Figure 7:**
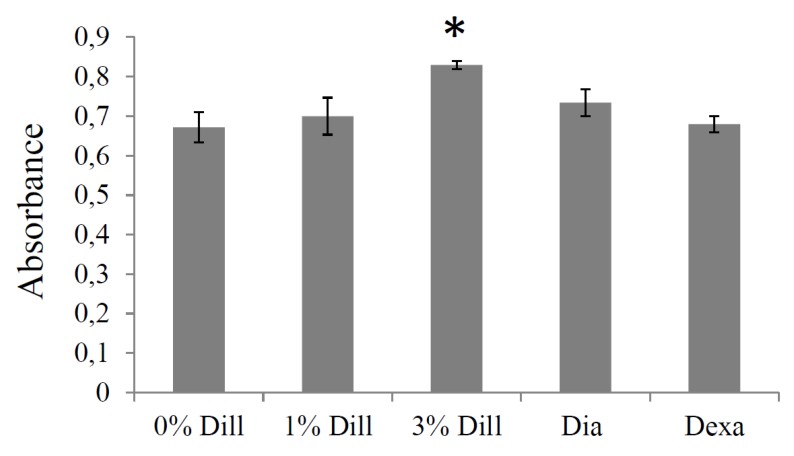
Effect of DE on elastin production by cultured vascular smooth muscle cells from the ascending aorta of aged mice. The effects of dexamethasone (Dexa, 0.1 µM) and diazoxide (Dia, 50 µM) were also tested as these compounds have previously been shown to result in elastin production stimulation by cultured aortic VSMCs (see text). Dill: DE. The absorbance is representative of extracellular elastin quantity. * Significantly different from 0% DE, the negative control (one-way ANOVA, followed by LSD tests, *p* ≤ 0.05). n = 5–6 in each group.

Acute treatment with 3% DE induced a significant increase in elastin synthesis (+24%) in cultured VSMCs from mouse ascending aortae. The 1% DE treatment, as well as dexamethasone and diazoxide at the concentrations tested [36,43], did not trigger a significant augmentation of elastin synthesis, although diazoxide produced a trend towards elevation (+10%; Figure 7).

## 4. Discussion

Identification of new therapeutic strategies to qualitatively and/or quantitatively improve the arterial wall elastic fibers could have major importance to counteract the age-associated alterations of blood vessel structure and function, and the cardiovascular system. In arteries or VSMC cultures from developing, adult, or aged rats or mice, several pharmacological treatments have shown promise to induce elastic fiber neosynthesis and/or elastin LOX or LOXL-1 expressions. However, several of those identified thus far, including the ATP-sensitive potassium channel openers and derivatives like minoxidil [4,29,30,31,32,33,34,35,36], have secondary effects such as edema and/or cardiac hypertrophy [4,31,33,36,44,45] that limit their usefulness. It was, therefore, important to continue the search for treatments devoid of such negative side effects. DE treatment has been shown, in vitro, to increase LOXL-1 gene expression in skin fibroblast and elastin production in dermal equivalent models [38], as well as to improve skin elasticity and smooth age-related face wrinkles in women [37]. In the present study, we have shown that a 3-month treatment of aged male mice with DE reduces the aortic elastic lamella disruptions induced by ageing (5% and 10% DE), leads to elastic fiber neosynthesis and functional improvement of the ascending aorta (10% DE), and reverses the age-related cardiac hypertrophy.

Most striking was the substantial protection of aortic elastic lamella integrity in aged mice produced by DE, with reductions by 23–33% of the number of elastic lamella disruptions observed after DE treatment. While the mechanism underlying this protective effect remains unclear, it may be related to the polyphenols, e.g., tannins and flavonoids that are present in dill seeds [46,47]. Polyphenols have previously been shown to have beneficial effects on the cardiovascular system through their antioxidant activity [48,49,50,51]. Additionally, polyphenols have been shown to dose-dependently reduce the activity of elastin-degrading enzymes belonging to the serine proteinase, cysteine proteinase, and metallo-proteinase families [52,53]. Finally, polyphenols have been shown to reverse age-related calcification of elastic fibers through inhibition of the activity of alkaline phosphatase [54,55].

Our results also indicated that 10% DE treatment increases the expressions of both tropoelastin and LOXL-1, two major contributors to elastic fiber synthesis and assembly in the aorta of aged mice. Additionally, we demonstrated that DE elevates the production of elastin by cultured VSMCs from the ascending aorta. Somewhat surprisingly, two previously demonstrated stimulators of elastin production by aortic VSMCs, dexamethasone and diazoxide [32,34,36] appeared less active in stimulating elastin biosynthesis in the ascending aortic VSMCs used in the present experiments, compared to that shown previously in cultured VSMCs from the entire aorta [32,34,36]. Here, the relative lack of VSMC responsiveness to these two molecules might be due to the differences in the developmental origin of the ascending aorta, used in the present experiments, and the aortic arch, in which the VSMCs arise from the neural crest, and the remainder of the aorta whose VSMCs arise from the mesoderm [56,57,58,59]. Supporting this hypothesis, aorta from these two different developmental origins have been shown to differentially respond to a variety of other activators. In embryos, compared to VSMCs from the descending aorta, VSMSCs from the ascending aorta or the aortic arch present substantially elevated α1 (I) procollagen, c-myb, integrin β8, and mesothelin mRNA levels in response to TGF-β1 stimulation and contract less in response to the vasoconstrictors KCl (at high concentration) and phenylephrine. Also, in adult mice, the ascending aorta constricts significantly more than the descending aorta in response to endothelin-1 [60,61].

We also observed numerous additional neo-synthesized elastic fibers of various orientations, particularly in radial fibers bridging adjacent elastic lamellae, in the aortic wall after DE treatment, particularly in 10% DE-treated aged mice, with no change in the number of elastic lamellae. Similarly, these same multiple orientations of neo-elastic fibers were observed after minoxidil treatment [33,36]. Notably, 10% DE treatment led to aortic wall thickening and subsequent decreased wall stress in aged mice, while 5% DE decreased aortic wall thickness, which tended to increase stress and return the stress–strain relation (i.e., circumferential forces applied within the wall to the cells and ECM at a given strain) closer to that in adult animals. The remodeling induced by 10% DE, not 5% DE, was accompanied by a significant increase in aortic distensibility and reduction of arterial stiffness in the physiological blood pressure range. The improved mechanical function of the aorta following 10% DE treatment of aged mice likely contributes to improved hemodynamics. This is of importance since aortic distensibility normally decreases with age, due to elastic fiber degradation, and is correlated with various cardiovascular diseases such as atherosclerosis, myocardial infarction, and high blood pressure [62,63]. Here, the improvement of arterial elasticity in the physiological pressure range by 10% DE could explain the observed reversal of the classical age-related cardiac hypertrophy, since LV mass is inversely correlated with aortic distensibility [64]. The decrease in the α-adrenergic-induced vasoconstriction induced by 10% DE treatment could also contribute to the reversal of age-related cardiac hypertrophy. In contrast, the reduction of blood pressure by 5% DE could account for a decrease in cardiac workload and explain the reversal of the age-related cardiac hypertrophy observed at this DE concentration. A possible role of the phenolic compounds present in dill seeds could also partly explain this effect of DE since treatment with the polyphenol resveratrol has been shown to prevent LV hypertrophy in obese mice [65]. The reversal of age-related cardiac hypertrophy by 5% and 10% DE treatments is a potentially important improvement compared to some previously characterized treatments that induce elastic fiber neo-synthesis such as minoxidil, which, in contrast to the effect of DE, result in increased heart weight and/or dimension in adult or aged animals [31,33,36]. It should be noted that DE-induced reversal of age-related cardiac hypertrophy without any significant impact on the body weight is essentially due to the reversal of the age-dependent hypertrophy of the left ventricle. The right ventricle weight was not impacted by ageing, and DE treatment maintained (i.e., did not decrease) its mass in aged animals. This again is an improvement compared to minoxidil, which elevates both the left and right ventricle masses in aged animals [33,36].

As noted above, an interesting aspect of the study related to the observation of some different responses of the ascending aorta to the 5% DE and 10% DE treatments. Many of the differences—with an effect of DE of higher amplitude or only observed with 10% compared to the 5% concentration—could simply be explained by a classical dose-response effect of DE on the VSMCs. This is the case for the effect of DE on elastin integrity (disruptions), the increase in TE and LOXL-1 mRNA levels, the neosynthesis of elastic fibers, the augmentation of the aorta wall thickness and related reduction of wall stress, the increased distensibility and decreased stiffness, and the responsiveness to phenylephrine. However, explanations for the observed effects induced by the lower concentration of DE, but not the higher concentration or opposite effects of the two concentrations, are less obvious. This is the case regarding the blood pressure reduction induced by 5% DE only, but not 10%, and the aortic wall thinning and subsequent trend towards increased stress induced by 5% DE (wall stress is inversely proportional to thickness), while 10% DE led to increased wall thickening and decreased stress. The wall thickness decrease in response to 5% DE could be explained by the reduction in blood pressure induced by 5% DE since it has long been established that long term blood pressure reduction and off-loading of the arterial wall leads to wall involution, i.e., atrophic wall remodeling, and decreased wall thickness [66,67]. However, the explanation for the finding that blood pressure is reduced in response to 5% DE, but not 10% DE, remains unclear. This could represent a differential effect of DE concentrations on the major cell types present in the aortic wall. A higher impact of 5% DE on endothelial cells, for example, could lead to an augmented production of NO, a major vasodilator, and decreased blood pressure. By contrast, higher DE concentrations, i.e., 10% DE, could result in differential stimulation of VSMCs, leading to increased vasoconstriction. Such a biphasic response involving endothelial cells at low dose and VSMCs at higher doses was also observed in the contractile responses of the aorta or coronary artery to acetylcholine and mesenteric artery to endothelin-3 [68,69,70]. Irrespective of this difference, however, both 5% and 10% DE resulted in elastic fiber preservation and neosynthesis, as well as functional improvements that led to reversal of the age-related left ventricular hypertrophy.

## 5. Conclusions

In conclusion, our results show that DE treatment of aged mice protects pre-existing elastic lamellae, reactivates tropoelastin and LOXL-1 expressions, induces elastic fiber neo-synthesis, and decreases the stiffness of the aging aortic wall, and results in reversal of the age-related cardiac hypertrophy. These findings strongly suggest that DE treatment improves cardiovascular function in aged animals. From this perspective, DE could be an important new anti-aging product for the cardiovascular system, which could be readily assessed in human clinical trials aimed at the prevention or treatment of age-related vascular stiffness and its sequellae. Identification of the active compound(s) present in DE and further pre-clinical experiments to determine optimal conditions leading to improved aortic elasticity and improvement of the orientations of the neo-elastic fibers generated by DE treatment, i.e., increasing the proportion of circumferentially-oriented neo-elastic fibers, would be beneficial. Studies evaluating the start of the treatment earlier in life (at 16–18 months of age) and/or an extension of the treatment duration could also be beneficial.

## Figures and Tables

**Table 1:** Body weight (BW) and ratios of total heart weight (HW/BW), left ventricle plus septum weight (LV + S/BW), and right ventricle (RV/BW) to BW in untreated adult mice as well as untreated and DE-treated (5% or 10% *v/v*) aged male mice.

	Adult		Aged	
		0% Dill	5% Dill	10% Dill
Body weight (g)	32.4 ± 1.5	30.4 ± 0.8	31.7 ± 0.8	32.5 ± 1
HW/BW	0.48 ± 0.01	0.56 ± 0.03 *	0.47 ± 0.003 ^&^	0.48 ± 0.03 ^&^
LV+S /BW	0.37 ± 0.006	0.43 ± 0.009 *	0.36 ± 0.005 ^&^	0.37 ± 0.02^&^
RV/BW	0.1 ± 0.004	0.12 ± 0.006	0.1 ± 0.002	0.1 ± 0.007

Data are mean ± SEM. n = 3–5 per group. Dill: DE. * Significant difference with the corresponding value in adult untreated mice (one-way ANOVA, followed by LSD test, *p* ≤ 0.05). ^&^ Significant difference between DE-treated and untreated aged mice (one-way ANOVA followed by LSD test, *p* ≤ 0.05).

**Table 2:** Histomorphometric analysis of the elastic lamellae in the ascending aorta wall.

	Adult		Aged	
		0% Dill	5% Dill	10% Dill
Number of elastic lamellae (EL)	6.81 ± 0.31	7.12 ± 0.36	7.58 ± 0.25	7.66 ± 0.33
Number of disruptions per EL	2.49 ± 0.11	5.93 ± 0.49 *	4.58 ± 0.2 *^&^	3.99 ± 0.46 *^&^

Data are mean ± SEM. Three animals per group were used. Dill: DE. * Significant difference with the corresponding value in adult untreated mice (one-way ANOVA, followed by LSD test, *p* ≤ 0.05). ^&^ Significant difference between DE-treated and untreated aged mice (one-way ANOVA followed by LSD test, *p* ≤ 0.05).

## Appendix A

### Surgical Procedure and Mechanical Studies

Animals were anaesthetized by intraperitoneal injection of pentobarbital (60 mg/kg), and an injection of heparin (90 units/30 g) was performed in the saphenous vein. The ascending aorta was quickly excised and placed in a physiological buffer (135 mM NaCl, 5 mM KCl, 1.17 mM MgSO_4_, 0.44 mM KH_2_PO_4_, 2.6 mM NaHCO_3_, 0.34 mM Na_2_HPO_4_,, 1.6 mM CaCl_2_-2H_2_O, 5.5 mM D-glucose, 0.025 mM EDTA, 10 mM HEPES; pH = 7.4). The vessels were cleaned of adhering connective tissue and fat, then cannulated and mounted onto a pressure arteriograph, as described previously [13,40]. The pressure arteriograph was composed of a plexiglass organ bath (filled with 10 mL physiological buffer), surrounded by a water jacket for heat regulation, in which two aligned cannulas (diameter: ~1 mm) are supported by holders. Each cannula was ligatured to one end of the ascending aorta, while the opposite end of the cannula was connected to a small tube joining a reservoir filled with physiological buffer, also allowing for changes of the pressure in the tubing and vessel. Verification of the absence of physiological buffer leakage (because of a wrong ligation or presence of a small hole in the vessel) upon pressurization of the vessel is then performed prior to starting the experiments. The experiments were performed at 37 °C and the physiological buffer present in the bath and in the vessel was replaced every 15 min. Following a 30-min equilibration period, the vessel was trans-illuminated under an inverted microscope connected to a charged-coupled device camera and to a computer. The proprietary software Windiam allowed for the continuous detection of the vessel inner and outer borders on the images from the camera (2 images/s) and recording of the vessel diameters [13]. Recordings of vessel inner diameters (IDs) and outer diameters (ODs) were taken while increasing the intravascular (transmural) pressure from 0 to 175 mmHg by steps of 25 mmHg (5 min per step). The inner diameter was directly measurable by transillumination above 125 mmHg, because of the thinning of the vessel wall due to increasing pressure-induced dilatation, allowing a clear and contrasted image of the inner wall edge. At lower pressures, as previously described, inner diameter can be accurately calculated through the entire range of pressure tested, from the measured OD and a unique measurement of vessel wall cross-sectional area (WCSA). As previously described [12,40], assuming that WCSA is constant, the formula finally used to calculate ID was: ID_x mmHg_ = √(OD^2^_x mmHg_ − OD^2^_175 mmHg_ + ID^2^_175 mmHg_)

Distensibility is defined as the change in relative volume (percentage) of the lumen per pressure unit during a change in intravascular pressure, as described by Smith and Kampine [41].

Distensibility (D), in percent, is: D = 100(ΔV/V_A_)/ΔP (A1)

V_A_ is the luminal initial volume before a small change in pressure. ΔV is the luminal volume change (V_A_ to V_B_) in response to a change in transmural pressure (from pressure A to B). The luminal volume of a given constant length (L) of a vessel segment is L.π.ID^2^/4, so that:

D = 100([L π Δ(ID_B_^2^/4)]/[L π ID_A_^2^/4])/ΔP (A2)

D = 100(Δ(ID_B_^2^)/ID_A_^2^)/ΔP = 100[(ID_B_^2^ − ID_A_^2^)/ID_A_^2^]/ΔP (A3)

Since the pressure change increment in our experiments was 25mmHg, we replaced, in the following sections, the distensibility (% change in volume per 1mmHg) by the distensibility per 25mmHg increment (D_25_). In any pressure range, a 25mmHg pressure change between pressure X mmHg and pressure X + 25 mmHg leads to the following expression of D_25_.

D_25_ = 100(ID_X+25_^2^ − ID_X_^2^) / ID_X_^2^ (A4)

Because the pressure-change increment in our experiments was 25mmHg, distensibility is expressed as the distensibility per 25mmHg increment (D25).

Circumferential midwall strain and circumferential wall stress were calculated according to the formulas given by Gibbons and Shadwick [42]:

Circumferential mid-wall strain (ε): ε = ΔR/R_0_ = (R − R_0_)/R_0_

Where R is the mid-wall radius (R = (OD + ID)/4) and R_0_ is the mid-wall radius at no transmural pressure (0mmHg).

Circumferential wall stress (σ): σ = P r/h

Where P is the pressure (in kPa), r is the luminal radius (=ID/2) and h is the wall thickness.

1 kPa is assumed to be equal to 7.5006mmHg.

*σ* represents the forces that are circumferentially applied on a each small portion (surface) of the vessel wall, because of the intraluminal pressure-induced stretching of the vessel.

Incremental elastic modulus (Einc): Einc = 0.75 (1+ε) Δσ/Δε

Einc is a measure of the wall stiffness. 1 kPa is assumed to be equal to 7.5006mmHg.

Einc represents the arterial wall stiffness.
